# Kinematic Plasticity during Flight in Fruit Bats: Individual Variability in Response to Loading

**DOI:** 10.1371/journal.pone.0036665

**Published:** 2012-05-15

**Authors:** Jose Iriarte-Diaz, Daniel K. Riskin, Kenneth S. Breuer, Sharon M. Swartz

**Affiliations:** 1 Department of Ecology and Evolutionary Biology, Brown University, Providence, Rhode Island, United States of America; 2 Department of Organismal Biology and Anatomy, University of Chicago, Chicago, Illinois, United States of America; 3 School of Engineering, Brown University, Brown University, Providence, Rhode Island, United States of America; Monash University, Australia

## Abstract

All bats experience daily and seasonal fluctuation in body mass. An increase in mass requires changes in flight kinematics to produce the extra lift necessary to compensate for increased weight. How bats modify their kinematics to increase lift, however, is not well understood. In this study, we investigated the effect of a 20% increase in mass on flight kinematics for *Cynopterus brachyotis*, the lesser dog-faced fruit bat. We reconstructed the 3D wing kinematics and how they changed with the additional mass. Bats showed a marked change in wing kinematics in response to loading, but changes varied among individuals. Each bat adjusted a different combination of kinematic parameters to increase lift, indicating that aerodynamic force generation can be modulated in multiple ways. Two main kinematic strategies were distinguished: bats either changed the motion of the wings by primarily increasing wingbeat frequency, or changed the configuration of the wings by increasing wing area and camber. The complex, individual-dependent response to increased loading in our bats points to an underappreciated aspect of locomotor control, in which the inherent complexity of the biomechanical system allows for kinematic plasticity. The kinematic plasticity and functional redundancy observed in bat flight can have evolutionary consequences, such as an increase potential for morphological and kinematic diversification due to weakened locomotor trade-offs.

## Introduction

Bats, like all mammals, experience both seasonal and daily changes in body mass. For example, during pregnancy, a female bat’s body mass can be up to 40% higher than during non-reproductive periods [1], [2], and during lactation, body mass may be even higher [3]. Similarly, both males and females of hibernating bat species experience changes in body mass as large as those observed in pregnant females [4]–[6]. On a daily scale, considerable variation in mass is associated with foraging, with changes of mass as large as 20–30% for insectivorous bats, 15–30% for nectarivorous bats [7], [8], and over 50% for sanguivorous bats [9]. Frugivorous bats often carry fruits as large as 40% of body mass to feeding roosts [10]. How these large changes in body mass affect kinematics and flight performance, however, is still poorly understood.

Over a wingbeat cycle of level flight at constant speed, a flying animal produces enough lift and thrust to counteract body weight and drag, respectively. Thus, any increase in body mass requires a proportional increase in lift to maintain level flight. Lift can be increased in multiple ways: by increasing the airspeed over the wings, by increasing the surface area of the wings, or by changing the three-dimensional wing configuration. Thus, it has been predicted that animals carrying a load can modulate lift generation by changing flight speed (e.g., [11]), wingbeat frequency and/or amplitude (e.g., [12], [13]), or the three-dimensional configuration of the wing such as angle of attack (e.g., [14]).

When body mass of flying vertebrates has been manipulated experimentally, no clear, consistent pattern of kinematic change results. For example, kestrels carrying loads of up to 30% body mass [15], [16] and insectivorous bats carrying loads up to 46% body mass [12] decrease flight speed and increase wingbeat frequency. In contrast, nectarivorous bats increase flight speeds in response to loading [7]. In ascending flight, individual *Cynopterus brachyotis* varied in their response to loading, but showed a tendency to increase wingbeat frequency and decrease wingbeat amplitude in loaded flights in which total power production was increased over the unloaded condition [17]. In other cases, responses have been complex, and animals adopted different strategies depending on the amount of load. With loads smaller than 15% body mass, cockatiels decreased their flight speed with no changes in wingbeat frequency, but at higher loads (i.e., 20% body mass), they increased both flight speed and wingbeat frequency [18]. These results suggest that the kinematic response to loading may not be straightforward, and that an individual may be able to select among multiple strategies for accommodating increased loading, depending on the magnitude of load and others factors, such as flight speed.

One challenge inherent in interpreting the results of studies carried out to date is that the effect of changes in flight speed cannot be decoupled from other changes in wingbeat kinematics, as kinematics change with speed as well as with loading (e.g., [19]–[22]). For example, it has been noted that wingbeat frequency tends to increase as speed decreases [23]. Thus, if a weighted bat decreases flight speed and increases frequency, the frequency increase could be the result of the increase in loading, the decrease in speed, or both. Furthermore, bats are also able to modulate their aerodynamic force generation by relatively subtle changes of their three-dimensional wing conformation and kinematics such as angle of attack, camber, and wing area, among others [24]–[26]. As a consequence, the three-dimensional kinematics of the body and wings provide a fuller and more nuanced view of how changes in mass affect flight in bats than less detailed overviews of flight behavior.

The aim of this study is to evaluate the effect of a substantial, transient increase in body mass on the three-dimensional kinematics of the lesser dog-faced fruit bat, *Cynopterus brachyotis,* across a range of speeds. We assessed detailed kinematics by employing animals trained to fly both in a wind tunnel, where speed was controlled, and in a flight corridor, where bats were free to select their flight speeds. An increase in aerodynamic force in response to loading can be achieved in multiple ways: i) by changing the force coefficient of the wings, which is a function of the three-dimensional wing configuration; ii) by changing the realized wing surface area, a function of the degree to which the joints of the wing are extended; or iii) by increasing the flow velocity over the wing surface, a function of flight speed, wingbeat frequency and wingbeat amplitude. We measured several wing shape and motion parameters, and predict that bats will employ some repeatable combination of these alternatives to increase aerodynamic forces in response to loading.

## Materials and Methods

### Animals and Loading Protocol

Three female lesser dog-faced fruit bats (*Cynopterus brachyotis*) (Table 1), loaned by the Lubee Bat Conservancy (Gainesville, FL) were subjects in this experiment. They were housed at the Harvard University-Concord Field Station (Bedford, MA), where they were provided with food and water *ad libitum*.

**Table 1:** Morphological measurements of the three individuals used in this study.

Variable	Individual		
	Bat1	Bat2	Bat3
Mass (kg)	0.0348	0.0371	0.0417
Wing span (m)	0.361	0.386	0.411
Wing area (m^2^)	0.0197	0.0212	0.0250
Aspect ratio	6.6	7.0	6.8
Wing loading (N m^−2^)	17.3	17.2	16.3

Measurements were performed following Norberg and Rayner [11].

**Figure 1:**
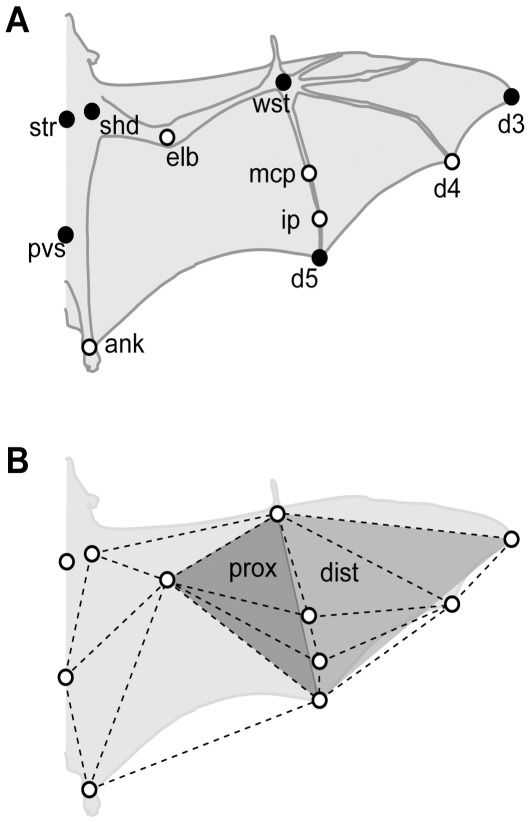
Markers and segmentation used in this study. Ventral view diagram of a bat indicating (A) the position of the wing and body markers and (B) the triangular segmentation used to calculate surface area, vertical force coefficient (*C*_v_), and angles of attack. The dotted lines indicate the 11 segments used to calculate surface area and *C*_v_ and the grey-shaded triangles represent the segmentation used to calculate the proximal (prox) and distal (dist) angles of attack. ank, ankle; d3, d4 and d5, distal end of of distal phalanx of digits III, IV and V, respectively; ip, interphalangeal joint of digit V; mcp, metacarpal-phalangeal joint of digit V; pvs, pelvis; shd, shoulder; str, sternum; wst, wrist. Black markers indicate the markers used in the flight corridor trials.

Before experiments, bats were anesthetized with isoflurane gas and key anatomical landmarks were marked with an array of high-contrast markers on the undersurface of one wing (Fig. 1A). All individuals experienced two treatments: *control*, in which there was no body mass modification, and *loaded*, in which body mass was increased by 20% (Table 2). Body mass was modified by injecting 0.9% saline solution into the peritoneal cavity, a technique that has been used to increase body mass in birds [27], in small terrestrial mammals [28], and in the same bat species used in this study [17].

**Table 2:** Body mass of experimental subjects for wind tunnel and flight tunnel corridor experiments, prior to the experiment, immediately after injection, and immediately after the end of the experiment.

	Individual		
	Bat1	Bat2	Bat3
**Flight corridor**			
original	35.53	36.62	42.36
after injection	42.49 (19.6%)	44.12 (20.4%)	51.06 (20.5%)
after experiment	42.01 (18.2%)	43.73 (19.4%)	50.67 (19.6%)
**Wind tunnel**			
original	34.87	37.42	41.10
after injection	41.82 (19.9%)	45.00 (20.2%)	49.29 (19.9%)
after experiment	41.65 (19.4%)	44.37 (18.6%)	48.96 (19.1%)

Body mass in g. Percentage of increase with respect to original mass appears in parenthesis.

Saline injection was performed while the bats were anesthetized. Subjects began to urinate immediately after awaking from anesthesia, so we provided fruit juice between trials to maintain body mass. Bats were weighed before and after every experimental session, which lasted <1 hr, to ensure that no substantial changes in mass had occurred (Table 2).

### Flight Experimental Setups: Flight Corridor and Wind Tunnel

The flight response of bats to increased loading was tested in two sets of experiments: one in still air (a flight enclosure), where bats were allowed to select their flight speed, and one in a wind tunnel, where flight speed could be experimentally controlled. In the flight corridor experiment, bats were trained to fly inside an enclosure (9 m long×1 m wide×2 m high). Bats were hand-released to fly from one end of the corridor to the other, and allowed to select their flight speeds. They also flew in the Harvard-Concord Field Station wind tunnel, an open-circuit tunnel with a closed jet in the flight chamber, and a working section 1.4 m long×1.2 m wide×1.2 m high (for technical details and aerodynamic characteristics see ref. [29]). Bats flew at different but overlapping speeds during flight corridor and wind tunnel trials. In the flight corridor, bats used speeds between 1.8 and 3.3 m s^−1^, and in the wind tunnel, speeds ranged from 3.1 to 8 m s^−1^. At speeds below 3.1 m s^−1^, bats did not maintain a steady position, but flew towards the front of the wind tunnel at greater than wind tunnel airspeed. The low speeds observed in the flight corridor are likely the effect of the enclosure itself, as it has been shown that in at least one species, bats tend to select lower flight speeds in shorter flight enclosures [7].

All components of this study were approved by the Institutional Animal Care and Use Committees at Brown University (#67-07), Harvard University (#27-10), and the Lubee Bat Conservancy (#CP07-2), and by the United States Air Force Office of the Surgeon General’s Division of Biomedical Research and Regulatory Compliance (#6F050).

### Three-dimensional Coordinate Mapping

Flight corridor trials were recorded at 500 frames per second using three high-speed Redlake PCI 1000 digital video cameras. The volume in which the bats were flown was calibrated using the Direct Linear Transformation (DLT) method, based on a 25-point (0.45×0.45×0.55 m) calibration cube recorded at the beginning of each set of trials [30]. Wind tunnel flights were recorded at 1000 frames per second using three high-speed Photron 1024 PCI digital cameras, calibrated by the DLT method with a 40-point (0.35×0.35×0.30 m) calibration cube, recorded at the beginning of each set of trials.

For the flight corridor trials, six markers on the bats’ bodies and wings were digitized from each video frame (*str*, *pvs*, *shd*, *wst*, *d3* and *d5* in Fig. 1A); for wind tunnel experiments, eleven markers were digitized (Fig. 1A). The three-dimensional position of each marker was resolved using the DLT coefficients obtained from the calibration cube. A 50 Hz digital Butterworth low-pass filter was used to remove high-frequency noise. This cut-off frequency, estimated by residual analysis [31], was approximately 5 times higher than the wingbeat frequency recorded in our bats.

### Kinematic Variables

A wingbeat cycle was defined by the vertical excursion of the wrist in a body coordinate system. Downstroke and upstroke phases were defined as the portions of the wingbeat cycle where wrist vertical velocities, relative to the body, were negative and positive, respectively.

#### Wing motion descriptors

Wingbeat frequency was defined as the inverse of the period between two consecutive upstroke-downstroke transitions. Wingbeat amplitude was defined as the angle between straight lines connecting the wingtip (*d3*) and the shoulder (*shd*) markers at the beginning and end of the downstroke. Stroke plane angle was defined as the angle between the horizontal axis and the least-squares regression line to the lateral projection of the wingtip during the downstroke [32].

Wingbeat frequency, downstroke ratio, wingbeat amplitude, stroke plane angle, and wingtip velocity were calculated from both flight corridor and wind tunnel experiments.

#### Wing configuration descriptors

Wing shape descriptors were calculated during the downstroke only for the wind tunnel experiments. The camber of the wing during downstroke was estimated by quantifying the curvature of digit V by fitting a parametric quadratic curve to the three-dimensional position of the four markers along that digit (*wst*, *mcp*, *ip*, *d5* in Fig. 1A). The fitted quadratic curve was then divided into 50 segments and the local curvature of each segment was calculated as the average rate of change in the tangent to the curve along its length [32], [33].

We measured the elbow and wrist joint angles to estimate the change in folding of the wing over the wingbeat cycle. Elbow joint angle was calculated as the three-dimensional angle between the shoulder, elbow and wrist markers (*shd*, *elb*, and *wst* in Fig. 1A), and the wrist joint angle as the three-dimensional angle between the elbow, wrist and wingtip markers (*elb*, *wst*, and *d3* in Fig. 1A).

To estimate changes in the realized wing surface area with changes in wing folding, we divided the wing into 11 eleven triangular elements (Fig. 1B) and calculated the area of each. Total wing area was obtained by multiplying this single wing area value by two. This value is necessarily smaller than the conventional value obtained from measurements of bats with wings completely extended over a flat surface because bats do not completely extend their wings during flight [24], [25] and because we do not include body area in this estimate.

We also designated triangular proximal and distal regions of the wing (Fig. 1B), and estimated angle of attack for each. Angle of attack was calculated as the angle between the vector of the relative incident air velocity and a plane formed by the three vertices of each region. The incident velocity vector was calculated as the first derivative of the position of the centroid of each triangle. It should be noted that our calculations do not account for induced velocity so we probably underestimate angles of attack, but we do not expect systematic changes in induced velocity between the control and loading treatments and therefore do not expect an effect on results.

#### Vertical force coefficient

The vertical force coefficient (*C*_v_) is a dimensionless number that depends, among other factors, on the angle of attack and camber of the wing, as well as on its velocity squared. Because of the flapping motion of the wings, more distal portions will move faster than proximal ones. Therefore, we divided one wing into 11 triangular elements (Fig. 1B), and for each of these segments we calculated surface area and velocity. We obtained the velocity of a segment by calculating the first derivative of the position vector of its centroid in the global coordinate system. We calculated the *C*_v_ during downstroke as:
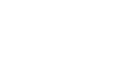
where *M*_b_ is body mass; *A*_v_ is the vertical acceleration of the center of mass; **g** is the acceleration of gravity, *ρ* is the air density, taken to be 1.2 kg m^−3^; *S_i_* and *V_i_* are the area and the velocity with respect to still air of the *i*-th triangular segment of the wing. The acceleration of the center of mass was estimated from a time-varying model of the mass distribution of the wing that accounts for wing kinematics [34]. Calculated in this way, *C*_v_ is not intended to represent an absolute measure but instead an assessment of relative aerodynamic effectiveness that is useful for comparisons across flight speeds and between loading treatments.

### Statistical Analysis

For all analyses, each bat was tested once at each speed, and we then calculated a representative value for each experimental trial as the mean of 3–5 wingbeats. Differences in kinematics in response to loading for flight corridor experiments were assessed with mixed-model analysis of variance (ANOVA) with individuals as a random effect. The effect of loading on wingbeat kinematics was estimated using analysis of covariance (ANCOVA), with loading as a fixed treatment and speed as a covariate. The linearity of the relationship with speed was estimated using a multiple regression approach with a quadratic speed component. If a variable did not change linearly with speed, or if the slope significantly differed between the unloaded and loaded treatments, the effect of loading was estimated by Tsutakawa’s Quick test [35]. All analyses were performed with JMP v.7, with a significance level of 0.05.

## Results

Wingbeat kinematics changed significantly in response flight speed and to loading (Table 3 and Table S1). However, each individual responded by modulating different combinations of kinematic parameters.

**Table 3:** Summary of kinematic changes for each individual in response to loading.

Variable	Individual		
	Bat1	Bat2	Bat3
Frequency (Hz)	↑	↓	↑
Amplitude (deg)	↓	↓	
Stroke plane angle (deg)			↓
Camber (m^−1^)	↑	↑	
Elbow extension (deg)	↑	↑	↑
Wrist extension (deg)	↑	↑	↑
Wing area (m^2^)		↑	
Vertical force coefficient, *C*_v_		↑	

Arrows represent significant positive (↑) or negative (↓) changes of a variable in response to 20% increase in body mass. Significance at α = 0.05.

**Figure 2:**
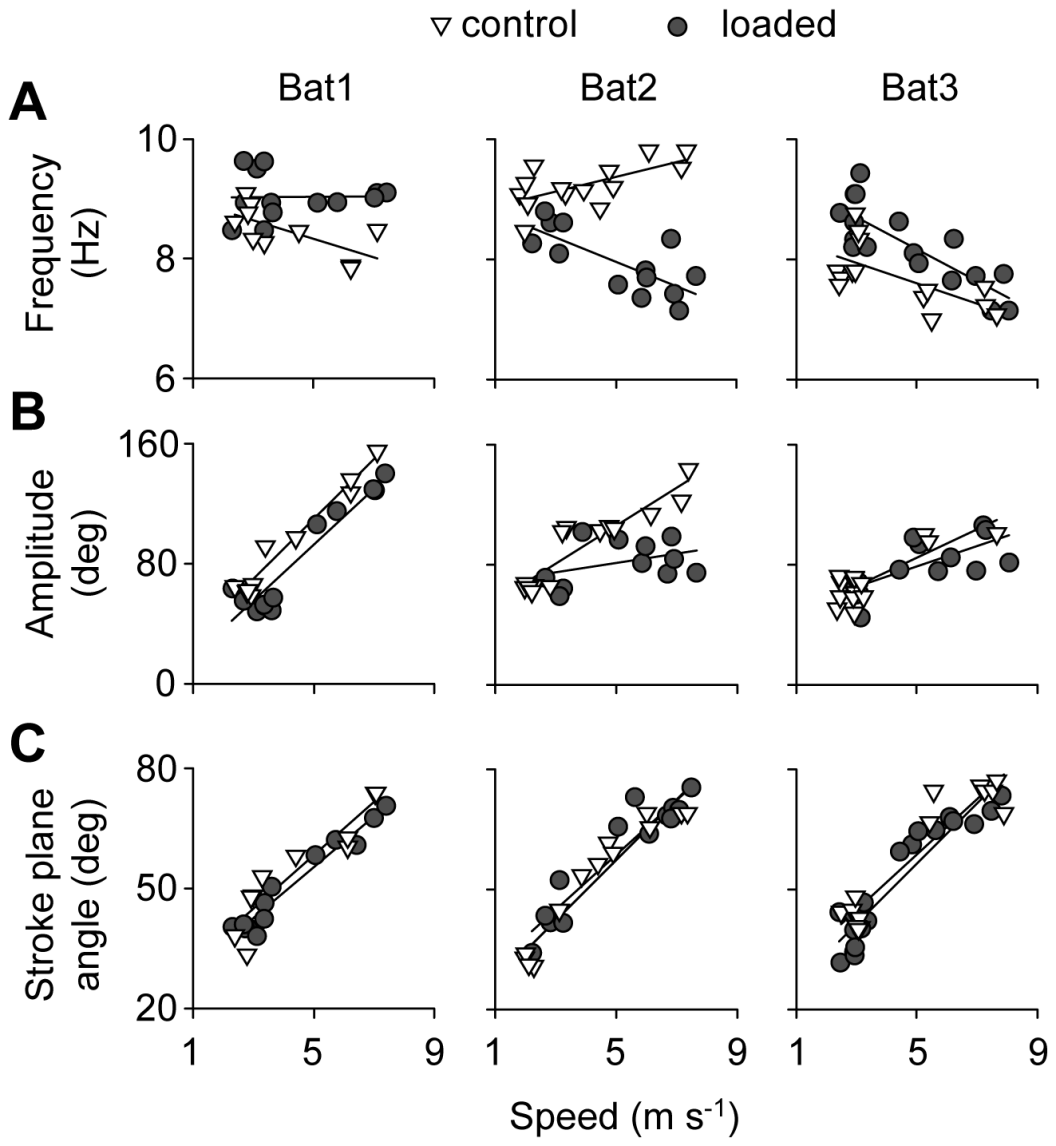
Wing motion parameters for bats in control and loaded conditions. Relationship between wingbeat frequency (A), wingbeat amplitude (B), and stroke plane angle (C) with flight speed. Open triangles represent control flights, grey circles represent loaded flights. Each point represents the mean value for a particular trial, using both wind tunnel and flight corridor flights.

**Figure 3:**
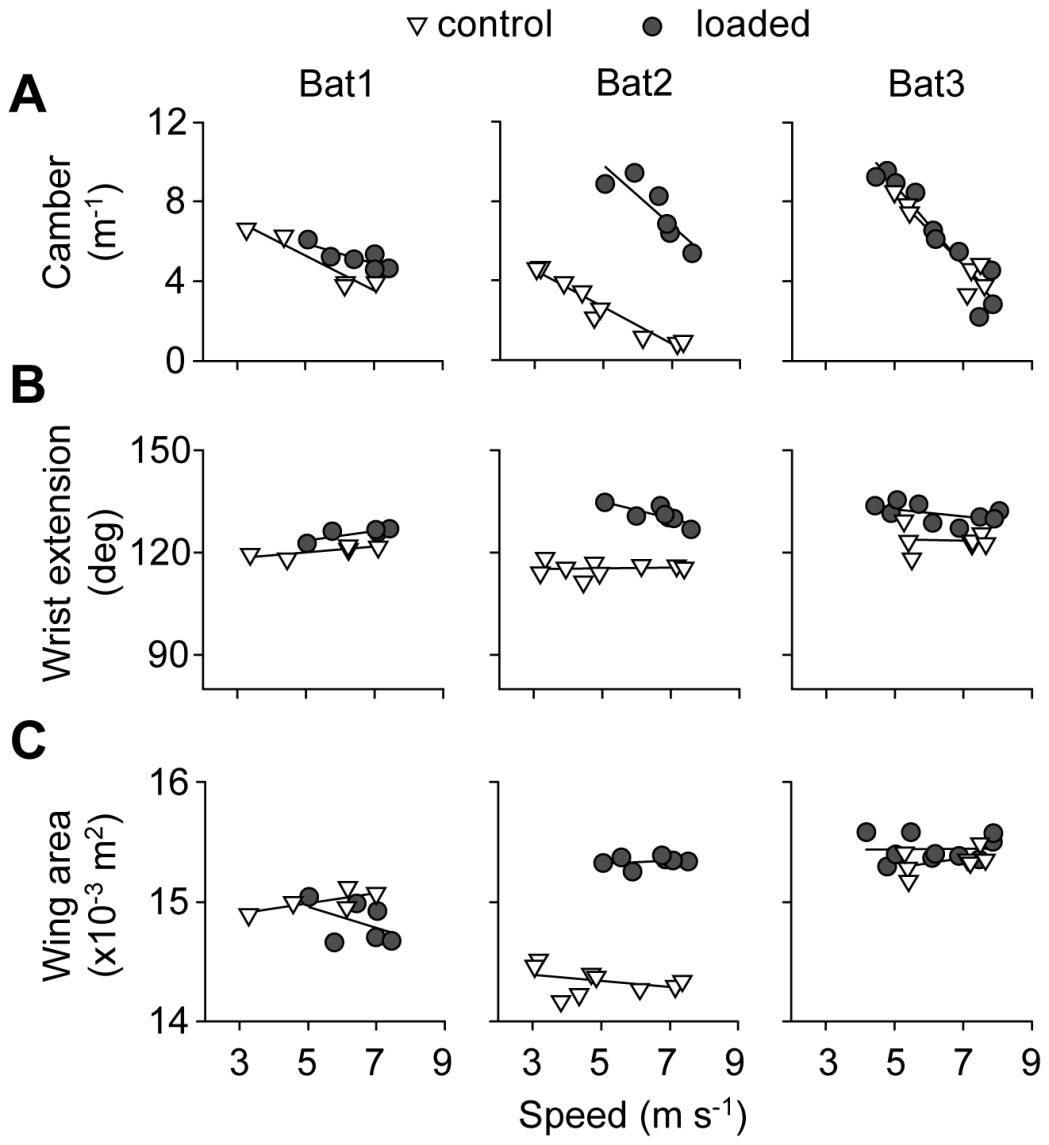
Wing shape parameters for bats in control and loaded conditions. Relationship between camber (A), wrist extension (B), and wing area (C) with flight speed. Open triangles represent control flights while grey circles represent loaded flights. Each point represents the mean value for a particular trial, using only wind tunnel flights.

**Figure 4:**
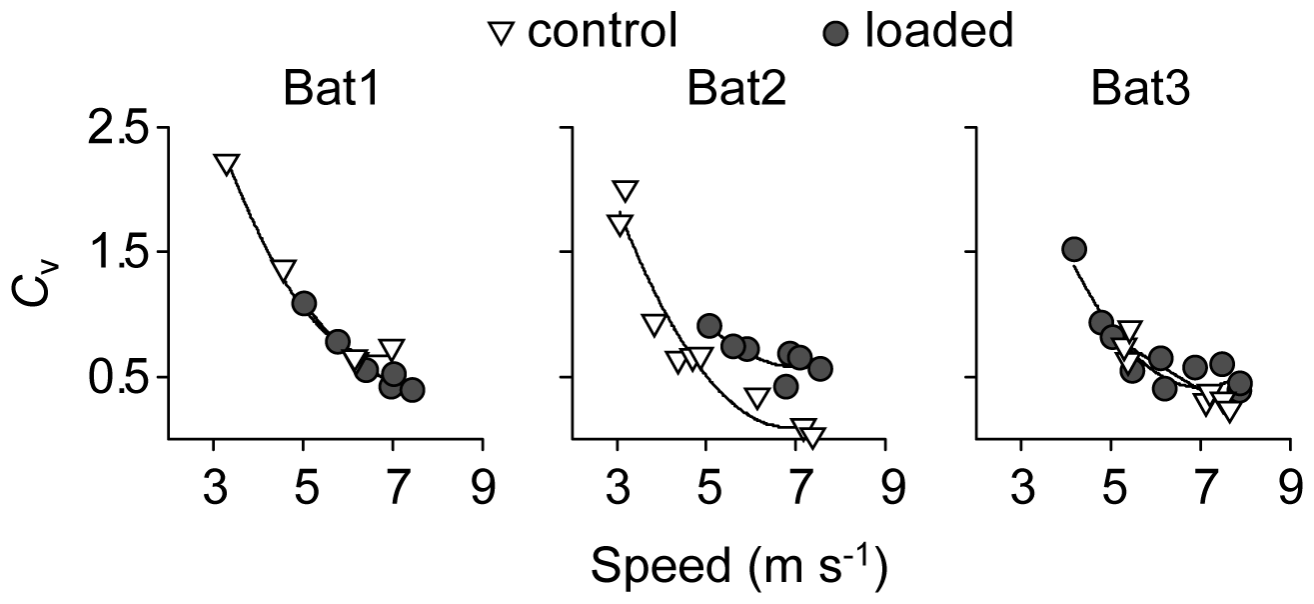
Vertical force coefficient for bats in control and loaded conditions. Relationship between the vertical force coefficient, *C*_v_, and flight speed for control (open triangles) and loaded (grey circles) flights. Each point represents the mean value for a particular trial, using only wind tunnel flights.

Kinematic patterns changed similarly with speed among individuals, in both control and loading conditions, with the exception of wingbeat frequency. Wingbeat frequency tended to decrease with flight speed, but it increased with speed for Bat2 in the control condition (Fig. 2A). In contrast, wingbeat amplitude and stroke plane angle increased similarly among individuals (Fig. 2B,C). In the same vein, kinematic parameters related to the three-dimensional configuration of the wing changed similarly with speed among individuals. Camber decreased linearly with speed (Fig. 3A), while neither wing extension nor wing area changed with speed (Fig. 3B,C). Finally, the vertical force coefficient decrease with speed, although not linearly, but in a similar fashion among individuals (Fig. 4).

In the flight corridor experiments, where bats were allowed to select their flight speeds, all individuals tended to fly faster with loading compared to the control condition, but this difference was not significant (one-way ANOVA, *F*_1,2.01_ = 9.6 *P* = 0.089). With increased loading, Bat1 increased wingbeat frequency and slightly decreased wingbeat amplitude (Fig. 2). Bat3 increased wingbeat frequency but also decreased stroke plane angle (Fig. 2). In contrast, Bat2 decreased both wingbeat frequency and wingbeat amplitude, in particular at high speeds (Fig. 2).

Individual bats varied in their modulation of the three-dimensional configuration of the wing in response to loading. Bat1showed small increases in camber, and elbow and wrist extension (Fig. 3). Bat3 also slightly increased elbow and wrist extension (Fig. 3B) but showed no significant change in wing area (Fig. 3C). Bat2, however, showed a very substantial increase in camber (Fig. 3A), elbow and wrist extension (Fig. 3B), as well as wing area (Fig. 3C). No significant changes in angle of attack were observed in any individual (Table S1).

Vertical force coefficient (*C*_v_) decreased with speed, and increased with loading only for Bat2 (TQT, *P* = 0.041; Fig. 4).

## Discussion

*Cynopterus brachyotis* showed a marked change in wingbeat kinematics in response to flight speed and to a 20% increase in body mass. The response, however, was non-uniform among individuals; each bat used a different kinematic strategy, varying different combinations of kinematic parameters to modulate force generation (Table 3).

### Individual Strategies of Kinematic Modulation

Wingbeat amplitude decreased with increased load in all bats, although this effect was marginally significant for Bat3. Similarly, major joints in the wing were more extended in the loaded flights in all bats. Outside of these consistent patterns, no two individuals responded to loading in exactly the same way. We can summarize the variation we observed, however, as two main strategies to increase vertical force generation: a ‘motion’ strategy, and a ‘shape’ strategy. Both Bat1 and Bat3 increased the flow over the wings by increasing wing speed, without significant changes in *C*_v_. Bat2 showed neither of these effects, but instead modulated the three-dimensional configuration of the wing, executing changes in camber and wing area, thereby increasing *C*_v_ and, accordingly, vertical force. The first strategy, henceforth called the motion strategy, requires that wingbeat kinematics change in a manner that results in greater airflow per unit time over the wings.

The second strategy, the ‘shape’ strategy, involved mainly the modulation of the three dimensional configuration of the wing. Bat2 showed substantially increased wing camber and wing area, and consequently, increased *C*_v_. Bat2 also modulated the motion of the wing, but did so in the opposite direction of predictions and of the behavior of the other subjects: both wingbeat frequency and amplitude decreased (Fig. 2A,B). Interestingly, Bat2 also showed a different kinematic response to speed. This bat increased flapping frequency as speed increased, in contrast to the other individuals that either decreased or did not change flapping frequency with speed. Kinematics are therefore plastic with regard to changes in speed as well as in response to loading.

It is plausible that if we were to increase sample size, we could find that other individuals would behave more like Bat1 and Bat3, and that Bat2 is an outlier and does not represent the typical kinematic response of *C. brachyotis*. However, a recent study on the flight kinematics of four *C. brachyotis* showed that there were consistent differences in wing motion among individuals and that every bat modulated its kinematics in a distinct manner in at least one kinematic parameter [21]. This suggests inherent individual variability, in this species at the very least, although we expect that this is a widespread phenomenon within bats.

But what is the nature of this variability? Are these different strategies specific and limited to each individual or can all individuals adopt them depending on the loading condition? A 20% increase in body mass is unlikely to be near the maximal loading capacity of fruit bats, considering that they have been observed carrying fruit of up to 40% their body mass [10]. Thus, if we were to increase loading conditions, we can expect at least two different scenarios: i) each bat keeps compensating by using the same observed strategies, either modifying the shape or the wing motion, or ii) they start incorporating alternative strategies (i.e., changing the shape and motion of the wings).

### Comparison with Other Flying Organisms

The responses to increased load that we observed in bats were complex, involving the modulation of both wing shape and wing motion. Wingbeat amplitude changed in a similar fashion among all individuals, tending to decrease with loading. This stands in direct contrast to other observations for flying animals. For example, loading experiments with hummingbirds have shown that wingbeat amplitude, along with small changes in wingbeat frequency, increases with loading [13], [36], [37]. Similarly, when hummingbirds are flown in low density air, a task that is functionally and mechanically similar to flying while carrying loads, they increase wingbeat amplitude to increase lift [37]–[42], as do several bee species [43], [44]. Interestingly, small specialist nectar-feeding bats hovering in low-density conditions show a similar response, with an increase in wingbeat amplitude, but no significant changes in wingbeat frequency [45]. But dog-faced fruit bats, the species we studied here, did not show a similar pattern of kinematic change in an ascending flight task [17]. Instead, these bats increased power production relative to the unloaded condition in only some flights, but when power production did increase, the basis for this increase was elevated wingbeat frequency and decreased amplitude.

### Individual Variation and Functional Equivalency

Individuals within a species may differ substantially in the kinematic strategies used to respond to loading. Although few studies have been specifically designed to measure individual differences in flight mechanics, variation among individuals of a species in kinematic patterns in response to an environmental or physiological challenge, demonstrated in this study, is not a phenomenon restricted to bats. Measurement of mechanical power output of pigeons carrying loads have shown large variations in the mechanical forces recorded for individual birds, indicating that response strategies to loading may differ among individuals [46]. Escape performance of naturally fattened great tits in preparation for migration demonstrates individual differences in flight speed [47]. Evidence of individual-specific flight strategies can also be found outside of experimental manipulation by addition of external loads. For example, there are individual differences in the control of body stabilization in sugar gliders [48] and also in the mechanisms of turning in Southern flying squirrels [49]. Although biologists have acknowledged the importance of individual variation in physiological, ecological, and evolutionary studies (see [50] for a review), it remains largely neglected in the study of animal flight. It has only been in recent years, as techniques and analyses became more and more automated, that larger numbers of individuals are being used and explicit measures of variability are analyzed (e.g., [51]–[53]).

The use of individual strategies by bats in our study resembles the concept of functionally equivalent systems (sensu [54]). Functionally equivalent systems are, in essence, complex systems that exhibit a pattern in which multiple combinations of underlying parts can give rise to emergent traits with similar mechanical, physiological, or performance values. Functional equivalence has been previously acknowledged in biological systems. For example, at the whole-organism level, morphologically different species can produce similar levels of biomechanical performance (e.g., [55]–[57]). Our results point to an additional layer of complexity that has not been fully appreciated previously, in which the inherent complexity of the biomechanical system allows for kinematic plasticity, i.e., functional equivalent kinematic responses, within and among individuals. This might be particularly true for bats. Bat wings possess more than two dozen joints with substantial independent control, and highly anisotropic, non-linearly elastic wing membrane with adjustable stiffness [58], [59], and an array of sensory organs hypothesized to provide local flow information during flight [60], [61]. Hence, unlike insects, and more even than birds, bats have the potentially to effect active, dynamic control over three-dimensional wing conformation, perhaps in response to local flow conditions on the wing [62]. Thus, there are multiple mechanisms a flapping flier with a highly articulated skeleton and wings of variable compliance can use to modulate the generation of aerodynamic forces.

Whole-organism performance represents the integration of numerous morphological, physiological, and behavioral traits. The complexity of the flight apparatus of bats allows multiple, redundant pathways of control to lead to similar levels of performance, which can have potential evolutionary consequences. Thus, selection may act on performance differently under specific ecological or physiological conditions, or may be constrained by the interactions of traits and/or functional trade-offs. Although this topic has yet to be investigated directly in the flight performance of bats, birds, or insects in natural settings, studies of locomotor performance in lizards have shown that while individual traits may not have direct effect on fitness, interactions with other traits and the environment can have important consequences on survival [63], [64]. Furthermore, a recent study has found evidence that complex functional systems can mitigate performance costs that result from competing demands on one trait (i.e., trade-offs) by compensatory changes in other traits [65]. Thus, the complexity of the flight apparatus may allow evolutionary changes in structure to be functionally neutral by producing compensatory changes in morphology and/or behavior, effectively increasing the range of usable kinematic configurations to generate a desire level of performance. If that is the case, complex systems may be characterized by flatter performance surfaces (i.e., with a larger combination of traits that yields maximum performance) than those of simple systems, and therefore making transitions between adaptive peaks more likely, and increasing the potential for morphological and functional diversification due to weakened trade-offs [65]. This is particularly suggestive considering that bats are the second most diverse group of mammals after rodents [66]. The differences in kinematic responses to loading that we found among individuals resembles the use of alternative escapes strategies used by some skinks, in which slower individuals preferred to dive underwater instead of running to escape predators [67]. Whether or not the differences observed in our bats represent discrete flight strategies (i.e., distinct peaks on the performance surface) or are part of a continuum of usable kinematics (i.e., a flat performance surface) remains an open question. As quantifying the highly complex kinematics of the bat wing grows simpler with technological advances (e.g., [68]), mapping kinematic performance surfaces will become more feasible with reasonable expenditure of time, and addressing this issue will soon be far more straightforward than in the past.

The results of this work highlight the importance of studying and reporting individual variation in natural and experimental conditions. If individual differences in kinematic strategies, such as those we observed in bats experiencing naturalistic loading, are widespread in flying organisms, studies of individual variability and how differences in kinematics map onto a kinematic-performance relationship can shed light on the underlying mechanistic basis of aerodynamic force generation and flight control.

## Supporting Information

Table S1 **Summary of ANCOVA analyses of kinematic variables in response to loading and speed.**
(PDF)
