# Supplementary material for: Kinematic Plasticity during Flight in Fruit Bats: Individual Variability in Response to Loading
Source: PLoS One. 2012 May 15;7(5):e36665. doi: 10.1371/journal.pone.0036665 (PMC3352941; doi:10.1371/journal.pone.0036665)
Supplement: Table S1 — Summary of ANCOVA analyses of kinematic variables in response to loading and speed. (PDF) [file pone.0036665.s001.pdf]

Table S1. Summary of ANCOVA analyses of kinematic variables in response to loading and speed.

	Bat1			Bat2			Bat3		
Variable	effect[1]	slope[2]	elevation[3]	effect	slope	elevation	effect	slope	elevation
Frequency (Hz)	↑	$F_{1,19}$=3.07	$F_{1,19}$=14.17**	↓	$F_{1,23}$=22.4***	TQT***	↑	$F_{1,28}$=1.81	$F_{1,28}$=19.8***
Amplitude (deg)	↓	$F_{1,19}$=0.21	$F_{1,19}$=14.1**	↓	$F_{1,21}$=11.4**	TQT*		$F_{1,26}$=2.80	$F_{1,26}$=3.55
Stroke plane angle (deg)		$F_{1,20}$=0.005	$F_{1,20}$=0.70		$F_{1,23}$=0.009	$F_{1,23}$=0.49	↓	$F_{1,31}$=0.31	$F_{1,31}$=10.8**
Camber (m^{-1})	↑	$F_{1,7}$=2.14	$F_{1,7}$=11.7*	↑	$F_{1,11}$=2.92	$F_{1,11}$=90.3***		$F_{1,13}$=0.53	$F_{1,13}$=0.14
Proximal AoA (deg)		$F_{1,6}$=4.2	$F_{1,6}$=0.82		$F_{1,14}$=0.0007	$F_{1,14}$=4.10		$F_{1,13}$=1.60	$F_{1,13}$=3.61
Proximal AoA (deg)		$F_{1,6}$=5.06	$F_{1,6}$=0.21		$F_{1,14}$=0.004	$F_{1,14}$=0.64		$F_{1,13}$=0.37	$F_{1,13}$=3.44
Elbow extension (deg)	↑	$F_{1,6}$=0.37	$F_{1,6}$=222.4***	↑	$F_{1,12}$=2.42	$F_{1,12}$=115.5***	↑	$F_{1,12}$=0.04	$F_{1,12}$=15.8**
Wrist extension (deg)	↑	$F_{1,6}$=0.69	$F_{1,6}$=26.1**	↑	$F_{1,12}$=5.70*	TQT*	↑	$F_{1,12}$=0.36	$F_{1,12}$=24.0***
Wing area (m^2)		$F_{1,7}$=2.66	$F_{1,7}$=3.08	↑	$F_{1,12}$=0.63	$F_{1,12}$=294.6***		$F_{1,13}$=0.72	$F_{1,13}$=4.04
C_v			TQT	↑		TQT*			TQT

[1]Effect represents the positive (↑) or negative (↓) change of a variable in response to loading.

[2]The equality of slope was determined by the interaction effect of 'loading' and 'speed' effects in an ANCOVA analysis.

[3]The elevation effect reflects the effect of the loading treatment over a variable and it was determined by ANCOVA analysis. If the slopes were different between unloaded and loaded treatments, significance was tested by a Tsutakawa's Quick test (TQT).

Statistical significance: $^{*}P<0.05$, $^{**}P<0.01$, $^{***}P<0.001$
